# Autophagy Inhibition Enhances the Anti-Tumor Activity of Methylseleninic Acid in Cisplatin-Resistance Human Lung Adenocarcinoma Cells

**DOI:** 10.3389/fphar.2022.890974

**Published:** 2022-05-03

**Authors:** Ming Xin, Qi Gao, Xindong Xiang, Juanjuan Xu, Yuhan Jiao, Xuan Li, Xianzhen Zhang, Xiuqin Jia

**Affiliations:** ^1^ The Key Laboratory of Molecular Pharmacology, Liaocheng People’s Hospital, Liaocheng, China; ^2^ College of Pharmacy, Shandong First Medical University, Taian, China; ^3^ Department of Oncology, Liaocheng People’s Hospital, Liaocheng, China

**Keywords:** lung adenocarcinoma, cisplatin-resistance, MSA, Akt/mTOR, autophagy, apoptosis

## Abstract

Cisplatin (DDP)-based chemotherapy remains one of the standard treatment options for patients with advanced lung adenocarcinoma (LUAD), and cisplatin resistance is the biggest challenge to this therapy. Autophagy is also closely associated with chemoresistance in LUAD. Desperately need to find a way to improve the treatment efficiency of cisplatin-resistant LUAD in clinical practice. Previous studies reported that methylseleninic acid (MSA) has good anti-proliferation and pro-apoptotic activities in tumor cells. However, the effectiveness of MSA on cisplatin-resistant LUAD and its effect on the induction of autophagy is still unclear. In the current study, we found that MSA effectively inhibited the proliferation of LUAD cell lines and triggered mitochondrial pathway-mediated apoptosis. This effect was more pronounced in cisplatin-resistant LUAD cells with high MDR1 expression. In contrast, the mitochondrial damage caused by MSA treatment can be degraded by inducing selective autophagy in LUAD cells, thereby exerting a self-protective effect on tumor cells. Mechanistically, MSA inhibits proliferation, promotes apoptosis, and induces autophagy in LUAD cells by inhibiting of the Akt/mTOR pathway. Combination with autophagy inhibitors reduces the effect of this selective autophagy-induced resistance, and thus enhancing even more the anti-tumor effect of MSA on cisplatin-resistant LUAD cells. Finally, We speculate that MSA in combination with autophagy inhibitors may be a promising new therapeutic strategy for the treatment of cisplatin-resistant LUAD.

## Introduction

As one of the most common malignancies in the world, lung cancer has claimed more lives than any other cancer (Brody, 2014). Approximately 85% of lung cancers are non-small cell lung cancer (NSCLC), and lung adenocarcinoma (LUAD) is the most common subtype of NSCLC (Han et al., 2019). Although some emerging treatment strategies have shown some curative effects (Yi et al., 2021), the prognosis of patients with malignant LUAD is still unsatisfactory, and the 5-years survival rate is about 10% or less (Zhao et al., 2018). Cisplatin (DDP) is the primary frontline basic drug for the treatment of various human malignancies including LUAD (Wu et al., 2015). However, cisplatin resistance is one of the major causes of chemotherapy failure and patient death in clinical treatment (Su et al., 2019). Therefore, it is still urgent to explore new methods to overcome the cisplatin resistance of LUAD.

Current researches have suggested that organic selenium compounds can increase the sensitivity of tumor cells to chemotherapy or radiotherapy, synergistically improving their therapeutic effects and reducing toxic side effects (Radomska et al., 2021). The high efficacy of these compounds has been observed in multidrug resistance (MDR) tumor cells, which brings hope to improve the therapeutic effect of tumor resistance (Tan et al., 2018). Methylselenic acid (MSA) is an organic selenium compound with the potential anti-tumor ability, which has been manifested in breast, prostate, pancreatic, lung and esophageal cancers (Wallenberg et al., 2014; Wang et al., 2021). MSA serves as the immediate precursor of methylselenol induces a variety of cellular biochemical reactions that are different from those generated by the selenium form converted by hydrogen selenide (Tarrado-Castellarnau et al., 2015). Researches have shown that MSA can enhance the effectiveness of multiple chemotherapeutic drugs, such as etoposide, cytarabine (Tan et al., 2018), paclitaxel (Qi et al., 2012), Irinotecan (Hu et al., 2005), and cyclophosphamide (Liu et al., 2016). It has recently been discovered that MSA can also make head and neck squamous cell carcinoma sensitive to radiotherapy. Therefore, MSA is a very potential anti-tumor drug, whether used alone or in combination with other chemotherapy and radiotherapy regimens. However, it is still unclear whether MSA has an effective on cisplatin-resistant lung adenocarcinoma.

Autophagy has been reported to have the ability to “prodeath” or “prosurvival” in cancer, which can resensitize chemotherapy drugs or enhance the chemotherapy resistance of tumor cells (Li et al., 2020). This ability may depend on the type and progression of cancer (Endo et al., 2017). Autophagy may promote the resistance of lung cancer cell to cisplatin, thereby playing a sustained role in protecting cells from cisplatin-mediated cytotoxicity (Wu et al., 2015). In addition, there are reports that MSA can induce autophagy in pancreatic cancer cells. However, whether MSA affects autophagy in cisplatin-resistant LUAD cells has not been fully elucidated.

This study aimed to explore the anti-tumor effect of MSA on cisplatin-resistant LUAD cells and whether MSA-induced autophagy has a protective effect on LUAD cells. Here, we proved that MSA inhibited the proliferation of cisplatin-resistant LUAD cells, triggered apoptosis mediated by the mitochondrial pathway, and induced autophagy, which may be achieved by inhibiting the AKT/mTOR signaling pathway. We also found that autophagy inhibition enhances the anti-tumor effect of MSA. MSA combined with autophagy inhibitors provides an innovative method for treating cisplatin-resistant LUAD.

## Materials and Methods

### Cell Culture

A549 cells and cisplatin-resistant A549/DDP cells were procured from Pituo Biological (Shanghai, China). Cells were cultured in modified RPMI medium (Cytiva, SH30809.01) supplemented with 10% fetal bovine serum (Abwbio, AB-FBS0500) in 5% CO_2_ at 37°C. To maintain the cisplatin resistance, A549/DDP cells were cultured with 2 μg/ml cisplatin, and the cisplatin was withdrawn 3 days before further study.

### Reagents

MSA (541,281) and dansylcadaverine (30,432) were purchased from Sigma-Aldrich (St. Louis, United States). Cisplatin was purchased from Qilu Pharmaceutical (Jinan, China). CQ (MB1668) and 3-MA (MB5063) were purchased from meilunbio (Dalian, China). SC79 (s7863) and MHY1485 (s7811) were purchased from Selleck (shanghai, China). The following antibodies were used, MDR1 (22336-1-AP), ALDH1A1 (60171-1-Ig), Bcl-2 (12789-1-AP), Bax (50,599-2-lg), Caspase-3 (19677-1-AP), Parkin (14060-1-AP), P62 (66,184-1-lg), LC3 (14600-1-AP), Akt (10176-2-AP), phospho-Akt (ser473) (66,444-1-lg), phospho-Akt (thr308) (29163-1-AP), mTOR (66,888-1-lg) were purchased from Proteintech (Wuhan, China). phospho-mTOR (ser2448) (#5536), GAPDH (#5174) were purchased from Cell Signaling Technology (Danvers, MA, USA). Horseradish peroxidase (HRP)-conjugated secondary antibodies (ab205718, ab205719) were procured from Abcam (Cambridge, United Kingdom).

### Western Blotting

Cells treated with drugs were lysed and harvested by RIPA lysis buffer (Beyotime, P0013) containing protease and phosphatase inhibitor (Beyotime, P1045). The protein concentration was determined by BCA protein assay kit (Beyotime, P0010). Quantitative protein samples were separated by SDS-PAGE (8–12%) and electrotransferred onto PVDF membranes (Millipore, ISEQ00010). The membrane was blocked with 5% milk for 1 h, incubated with the designated primary antibody for 24 h at 4°C, and then incubated with the adapted HRP-conjugated secondary antibody for 1 h at room temperature. Subsequently, the reaction was performed with an ECL chemiluminescence kit (Millipore, WBKLS0100), and the protein bands were visualized and analyzed by the chemiluminescence system (Tanon, 5200Multi).

### Cell Viability Analysis

Cells were treated with a specific dose of MSA and/or CQ, 3-MA for 24 h, then 10% CCK-8 reagent (Beyotime, C0039) was added and incubated at 37°C for 2 h. The OD was measured at 450 nm with a microplate reader (Thermo, Multiskan FC). The cell viability was calculated according to the following formula:

$$\mathrm{cell}\;\mathrm{viability}\left(\%\right)=\frac{\mathrm{OD}experiment\;-\mathrm{OD}blank}{\mathrm{OD}control\;-\mathrm{OD}blank}\times 100\%$$

### Colony Formation

Cells were seeded into a 6-well plate with 1,000 cells per well for 24 h. Cells were treated with MSA for 14 days, during which the medium was changed every 3 days. Then the cells were fixed with 4% paraformaldehyde for 0.5 h, stained with 0.1% crystal violet (Solarbio, G1063) for 15 min, and the survival rate of the clonogenic was counted.

### Flow Cytometry Analysis

Cells were digested and collected by trypsin without EDTA after drug treatment. Each sample was added 5 μL Annexin V-FITC and PI (BD, 556,547) for 15 min in the dark. The apoptotic ratio was analyzed by flow cytometry (BD, LSRFortessa). Apoptosis was calculated as the ratio of early (Q4, Annexin V^+^/PI^−^) apoptotic cells. It is noted that late apoptotic cells (Q2) were excluded from the quantification, as they cannot be reliably distinguished from necrotic cells by this assay.

### TUNEL Analysis

Cell slides were treated with drugs for 24 h, fixed with 4% paraformaldehyde for 0.5 h and permeabilized in 0.1% Triton X-100 for 5 min. Each cell slide was stained by adding 50 μL TUNEL (Beyotime, C1088) detection solution, and used the antifade mounting medium with DAPI (Beyotime, P0131) for mounting. Observed the apoptosis through the fluorescence microscope (Nikon, Ti2-U).

### JC-1 Analysis

Cells were treated with MSA for 24 h, 0.5 ml of JC-1 (Solarbio, M8650) was added and incubated 37°C for 20 min. The changes of mitochondrial membrane potential (MMP, ΔΨm) were observed by a fluorescence microscope (Nikon, Ti2-U).

### MDC Analysis

After MSA treatment, equal numbers of cells were harvested and stained with 10% MDC for 45 min at 37°C and visualized with a fluorescence microscope (Nikon, Ti2-U).

### Transmission Electron Microscope Observation

Cells were collected and fixed in 0.1 mol/L glutaraldehyde at 4°C for 24 h, and then they were wrapped in the agarose and fixed with 1% osmium tetroxide (pH 7.4) for 2 h. Each sample was dehydrated, embedded, polymerized, ultrathin sectioned, and then used 2% uranium acetate saturated alcohol solution and 2.6% Lead citrate to avoid CO_2_ staining for 8 min. The ultrastructure of cells was observed by TEM (HITACHI, HT7800).

### Confocal Laser Scanning Microscopy and Image Analysis

Cells were seeded in a 6-well plate with 5 × 10^4^ cells per well for 24 h. When the cells confluence was 30%, they were infected with the constructed mRFP-GFP-LC3 lentivirus (Genechem, MAP1LC3B). The optimal infection conditions were 10 μL lentivirus (10^8^ TU/mL) + 40 μL HitransG P (25×) + 950 μL complete medium. After 16 h of infection, the cell culture was changed to a complete medium and continued to culture for 72 h. The infection efficiency was observed and selected with 2 μg/ml puromycin (Abcam, ab141453). After ensuring that the cell lines stably expressed mRFP-GFP-LC3 were constructed, cells were treated with drugs for 24 h. Antifade installation media with DAPI was used to stain the cell nucleus, and acquired the image with confocal laser scanning microscopy (CLSM, Leica, TCS SP5).

### Statistical Analysis

All data are displayed as the mean ± SD of at least three independent experiments. Student’s t-tests were compared between two groups. Comparisons between multiple groups were assessed by two-way ANOVA, and multiple comparisons were performed using Graphpad Prism. The *p*-value for data analysis was less than 0.05, which means it is statistically significant.

## Results

### Methylseleninic Acid Reduced the Proliferation of Cisplatin-Resistant Lung Adenocarcinoma Cells

**FIGURE 1:**
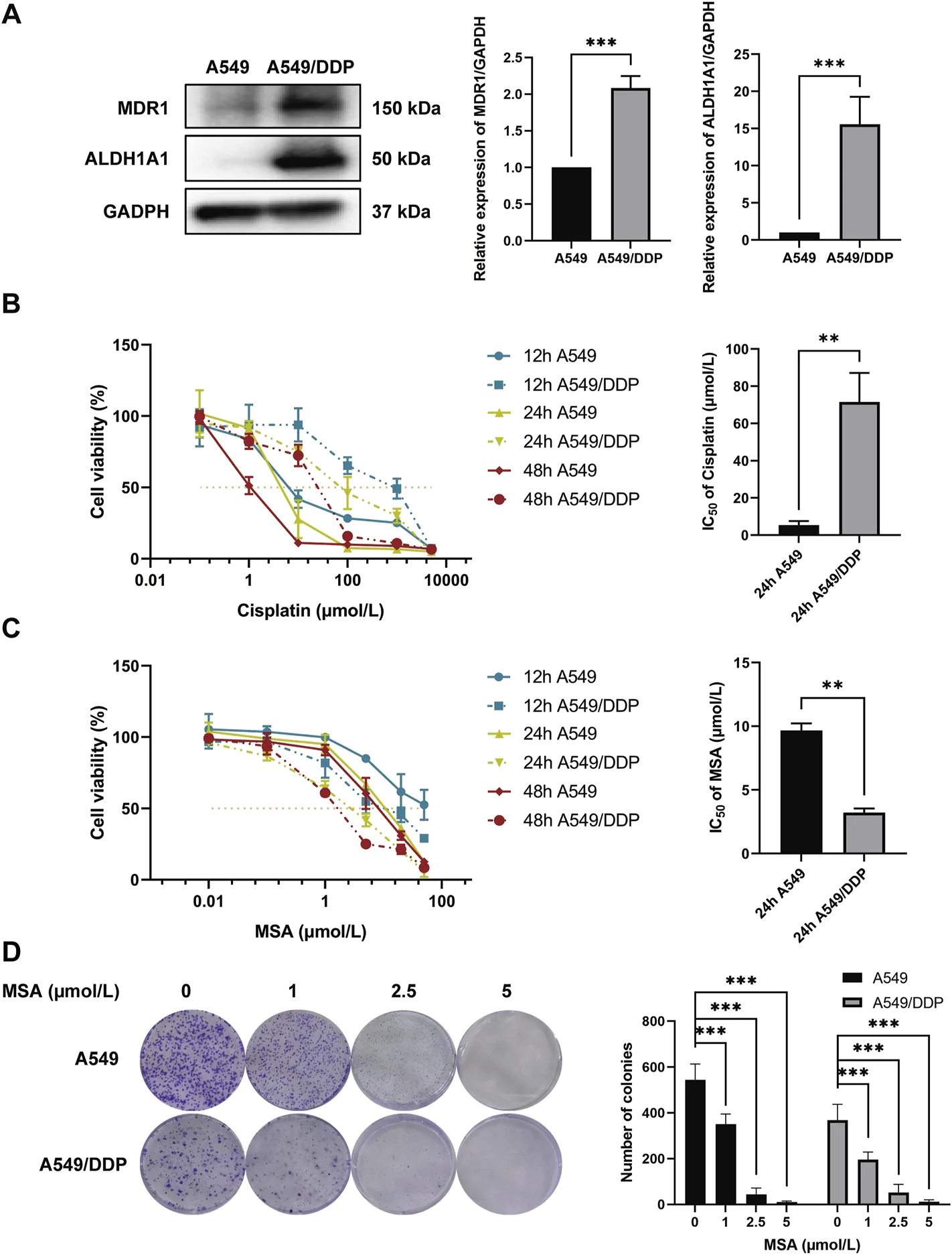
MSA reduced the proliferation of cisplatin-resistant LUAD cells **(A)** The protein expression levels of MDR1 and ALDH1A1 in parental A549 cells and cisplatin-resistant A549/DDP cells was evaluated by western blotting **(B,C)** Cells were treated with different gradient concentrations of cisplatin and MSA for 12, 24 and 48 h. The cell viability was determined by CCK8, and the IC50 value was quantified **(D)** Colony formation assay was used to detect the effect of MSA treatment on the proliferation of A549 and A549/DDP cells. Surviving cell colonies were stained with crystal violet and statistically analyzed for clone formation. Values are expressed as mean ± SD, *n* = 3. *p < 0.05, **p < 0.01, ***p < 0.001 compared with the control group.

To determine whether A549/DDP cells used in this study are resistant to cisplatin, we first detected the expression level of MDR1 and ALDH1A1 in A549 and A549/DDP cells (Figure 1A). As the results showed, compared with the parental A549 cells, the expression of MDR1 and ALDH1A1 protein in A549/DDP cells were significantly increased. The cell viability of A549/DDP cells was higher than that of A549 cells when treated with cisplatin for 12, 24 and 48 h. Moreover, A549/DDP cells were approximately 13-fold more resistant to cisplatin than A549 cells (IC_50_ of cisplatin for 24 h treatment was 71.51 ± 15.59 versus 5.39 ± 2.18 μmol/L) **(** Figure 1B **)**. The effect of MSA on the proliferation of A549 and A549/DDP cells was subsequently examined, and MSA treatment reduced the cell viability of A549/DDP cells compared to A549 cells after 12, 24 and 48 h. The IC_50_ of MSA in A549 and A549/DDP cells treated for 24 h were 9.67 ± 0.78 μmol/L and 3.22 ± 0.46 μmol/L, respectively **(** Figure 1C). Cisplatin-resistant A549/DDP cells are more sensitive to MSA. In addition, the colony formation assay demonstrated that MSA inhibited the colony formation of A549 and A549/DDP cells in a dose-dependent manner **(** Figure 1D).

### Methylseleninic Acid Induced Mitochondrial-Mediated Apoptosis in Cisplatin-Resistant Lung Adenocarcinoma Cells

**FIGURE 2:**
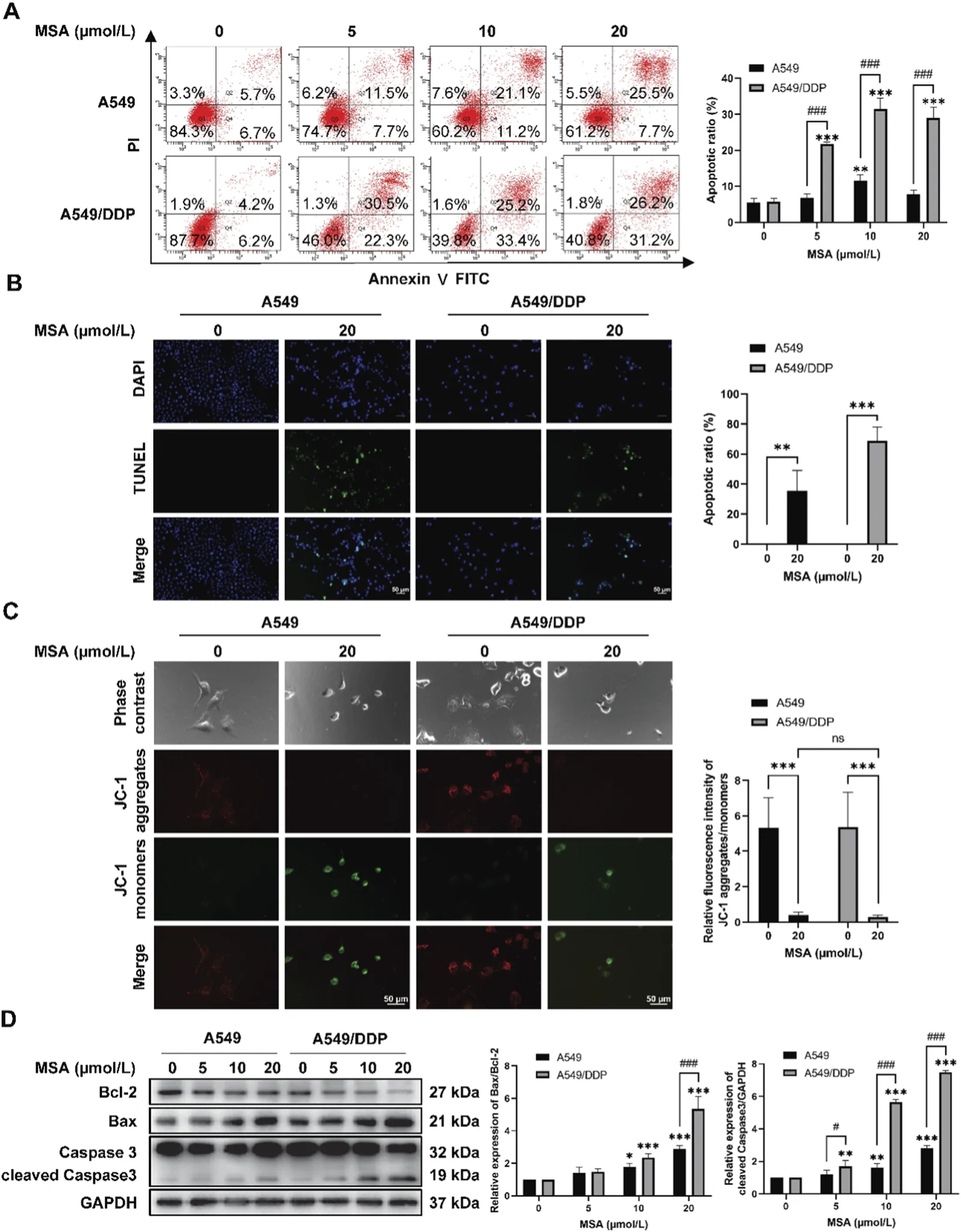
MSA induced mitochondrial-mediated apoptosis in cisplatin-resistant LUAD cells. **(A)** Flow cytometry analysis was used to examine the early apoptotic ratio (Annexin V^+^/PI^−^ cells, Q4) in A549 and A549/DDP cells after MSA treatment. **(B)** Apoptosis was determined by observing and counting TUNEL and DAPI stained cells under fluorescence microscopy. Scale bars: 50  μ. **(C)** Changes in MMP were observed by JC-1 staining under fluorescence microscopy. Scale bars: 50  μ. **(D)** The protein expression levels of Bcl-2, Bax, Caspase-3, and cleaved Caspase-3 were detected by Western blotting, and the relative protein expressions were shown as bar graphs after normalized analysis. *p < 0.05, **p < 0.01, ***p < 0.001 vs. control group (untreated cells), #*p* < 0.05, ###*p* < 0.001 indicated that A549/DDP group compared to A549 group under the same concentration of drug treatment (*n* = 3).

To determine whether the anti-tumor activity of MSA in cisplatin-resistant LUAD cells is related to apoptosis, we observed the effects of MSA on apoptosis in A549 and A549/DDP cells by Annexin V FITC/PI and TUNEL staining. After A549 and A549/DDP cells were treated with MSA for 24 h, the apoptotic rate increased in a dose-dependent manner, and under the same concentration of MSA treatment, more apoptosis was induced in cisplatin-resistant A549/DDP cells **(** Figure 2A **)**. The results of TUNEL staining also confirmed that MSA induced apoptosis of A549 and A549/DDP cells, which were consistent with the results of flow cytometry analysis (20 μmol/L) **(** Figure 2B). Since the reduction of MMP was associated with the apoptotic in cells, we examined the effect of MSA on the integrity of the mitochondrial membrane by JC-1 staining. The results showed that the relative fluorescence intensity of JC-1 aggregates/monomers decreased in A549 and A549/DDP cells treated with MSA for 24 h compared to the control group, However, there are no significant differences in MMP between the 2 cells when they responded to the MSA treatment.**(** Figure 2C). It is suggested that MSA may induce LUAD cells apoptosis by affecting mitochondria function. Subsequently, we tested the essential indicators of mitochondrial-mediated apoptosis, Bcl-2, Bax, and Caspase-3, and cleaved Caspase-3 by western blotting. After MSA treatment, the level of Bax/Bcl-2 increased in a dose-dependent manner. Compared with A549 cells, the change of Bax/Bcl-2 was more obvious in A549/DDP cells. Simultaneously, cleaved Caspase-3 were increased in the same manner, and this change was more pronounced in A549/DDP cells with the increase MSA concentration **(** Figure 2D). These results showed that MSA treatment can induce mitochondrial-mediated apoptosis in cisplatin-resistant LUAD cells.

### Methylseleninic Acid Induced Autophagy in Lung Adenocarcinoma Cells

**FIGURE 3:**
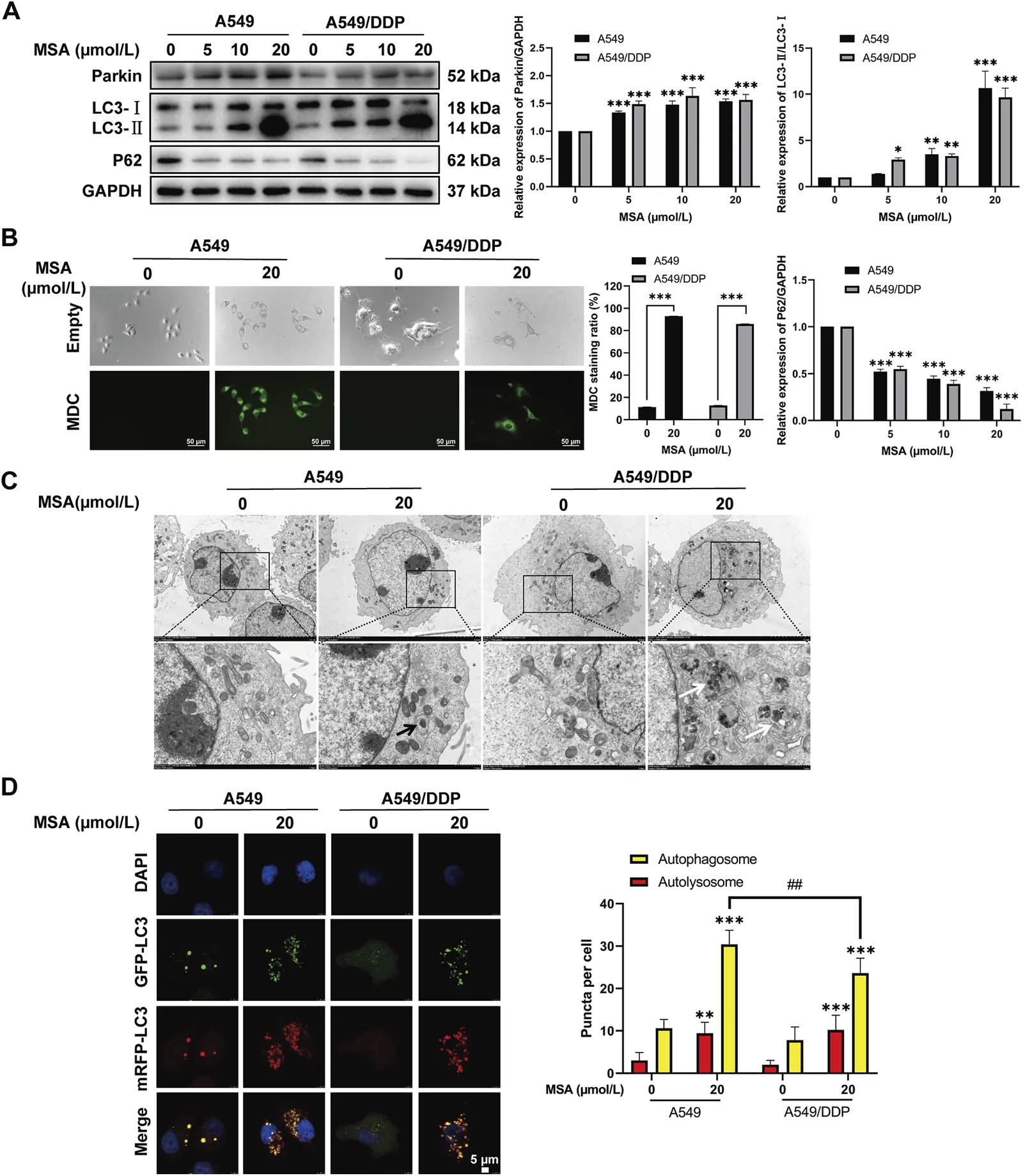
MSA induced autophagy in LUAD cells **(A)** The protein expression levels of Parkin, P62, and LC3 in A549 and A549/DDP cells after MSA treatment were detected by western blotting, and the data were normalized for quantitative analysis **(B)** The ratio of positive cells to form autophagic vesicles induced by MSA was observed by MDC staining under fluorescence microscope. Scale bars: 50 μm **(C)** Observation of autophagosomes (black arrows) and autolysosomes (white arrows) formed in cells after MSA treatment by TEM. Scale bars: 5 μm **(D)** Changes in autophagosomes (yellow puncta) and autolysosomes (red puncta) in cells stably expressing mRFP-GFP-LC3 after drug treatment were detected by CLSM. Representative images are shown. Scale bars: 5 μm *p < 0.05, **p < 0.01, ***p < 0.001 vs. control group (untreated cells), ##*p* < 0.01 indicated that A549/DDP group compared with A549 group under the same concentration of drug treatment (*n* = 3).

Since the decrease of the MMP will cause the damage of mitochondria, recruit and activate the biological activity of Parkin. Eventually, the damaged mitochondria are engulfed by autophagosomes and degraded by autolysosomes (Nguyen et al., 2016). MSA treatment increased Parkin expression in a dose-dependent manner, except for MSA (20 μmol/L) treatment in A549/DDP cells. Levels of the autophagosome marker LC3-II/I were increased after MSA treatment in a dose-dependent manner, the autophagy receptor P62 was decreased in the same way. **(** Figure 3A **)**. MDC can selectively fluorescently label autophagic vacuoles. The results revealed that the ratio of MDC-stained positive cells was increased in both A549 and A549/DDP cells after MSA treatment **(** Figure 3B). Then we observed the increase of autophagosomes (black arrows) and autolysosomes (white arrows) in A549 and A549/DDP cells by TEM after MSA treatment **(** Figure 3C). To determine the effect of MSA on autophagic flux, we used lentivirus transfection to construct A549 and A549/DDP cells that stably express mRFP-GFP-LC3. Autophagic flux were monitored by detecting autophagosomes (mRFP/GFP colocalization, yellow puncta) and autolysosomes (mRFP only, red puncta) using a CLSM (Hieke et al., 2015). Consistently, MSA treatment increased the number of autophagosomes and autolysosomes compared to controls, indicating that the autophagic flux in A549 and A549/DDP cells increased. However, fewer autophagosomes were induced in A549/DDP cells after MSA treatment compared to A549 cells **(** Figure 3D).

### Methylseleninic Acid Inhibited the Akt/mTOR Signaling Pathway

**FIGURE 4:**
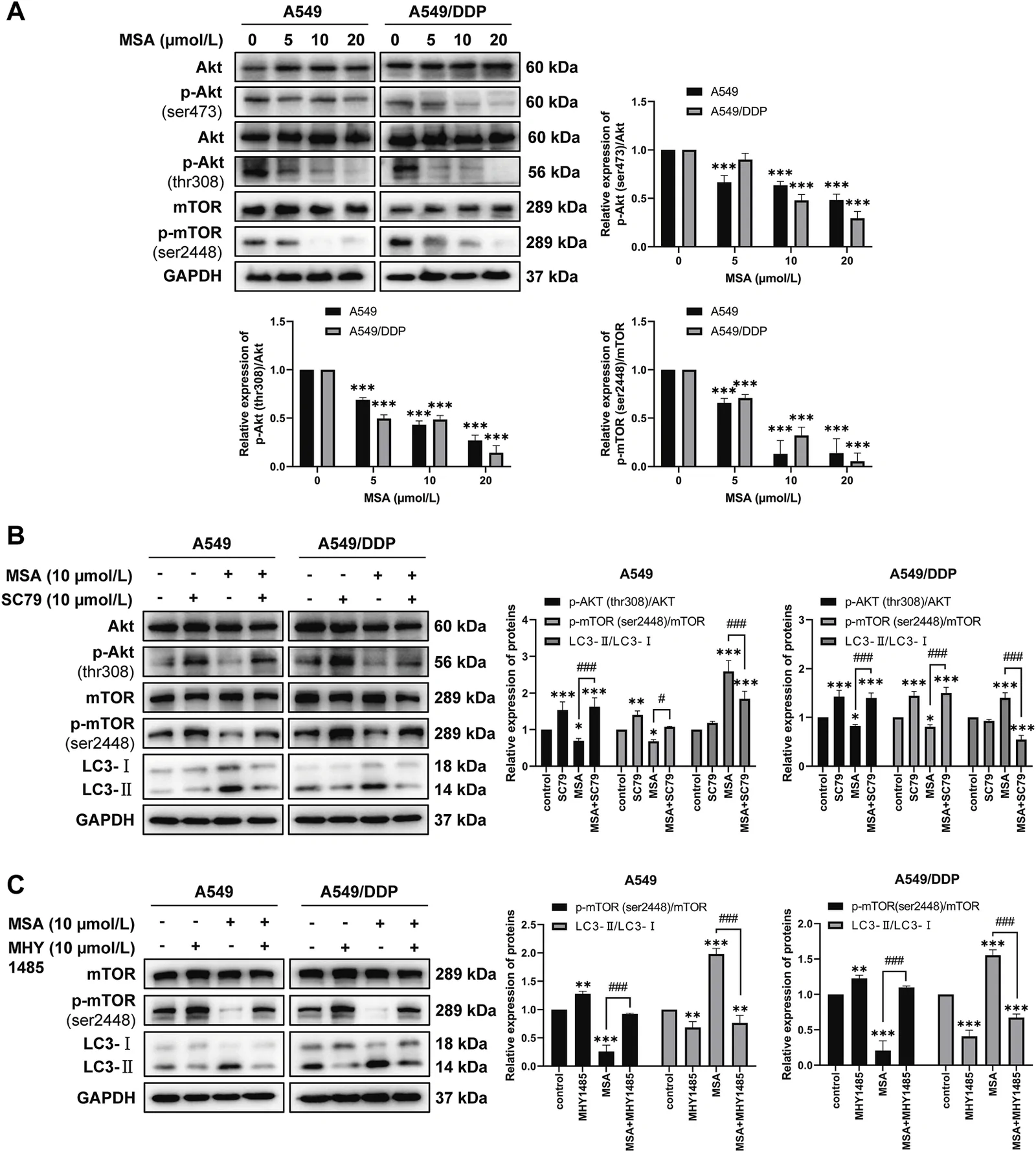
MSA inhibited the Akt/mTOR signaling pathway **(A)** The protein expression levels of Akt, p-Akt (ser473), p-Akt (thr308), mTOR and p-mTOR (ser2448) in MSA-treated A549 and A549/DDP cells were detected by western blotting **(B)** A549 and A549/DDP cells were treated by MSA (10 μmol/L) implement with or without SC79 (10 μmol/L) for 24 h, respectively. Akt, p-Akt (thr308), mTOR, p-mTOR (ser2448) and LC3 were detected by western blotting **(C)** Cells were treated by MSA (10 μmol/L) implement with or without MHY1485 (10 μmol/L) for 24 h, respectively. mTOR, p-mTOR (ser2448) and LC3 were detected by western blotting. Data were normalized for quantitative analysis and presented by bar graph. *p < 0.05, **p < 0.01, ***p < 0.001 vs. control group (untreated cells), #*p* < 0.05, ##*p* < 0.01, ###*p* < 0.001 indicated that SC79 or MHY1485 combined with MSA treatment versus MSA single treatment group (*n* = 3).

To further elucidate the underlying molecular mechanism of MSA promoted apoptosis and induced autophagy, we analyzed the protein expression level of total Akt, mTOR that the central upstream regulator of autophagy, the phosphorylation of Akt at ser473 and thr308 as well as the phosphorylation of mTOR at ser2448. After MSA treatment, p-Akt (ser473), p-Akt (thr308) and p-mTOR (ser2448) were reduced in a dose-dependent manner **(** Figure 4A **)**. To further detect whether the induction of autophagy by MSA is mediated through suppressing of the AKT/mTOR pathway. SC79 (an activator of AKT) and MHY1485 (an activator of mTOR) were used to activate the AKT/mTOR pathway upon MSA treatment to see whether it can abolish MSA induced autophagy. Compared with MSA treatment alone, co-treatment with MSA and SC79 significantly increased the expression of p-AKT (thr308) and p-mTOR (ser2448), whereas reduced the expression of LC3-II/I in A549 and A549/DDP cells **(** Figure 4B). Meanwhile, co-treatment with MSA and MHY1485 also increased the expression of p-mTOR (ser2448) and decreased the expression of LC3-II/I (Figure 4C). The above results indicated that the autophagy process was inhibited when compared with MSA treatment alone.

### The Effect of Methylseleninic Acid-Induced Autophagic Flux Was Blocked by Autophagy Inhibitors

**FIGURE 5:**
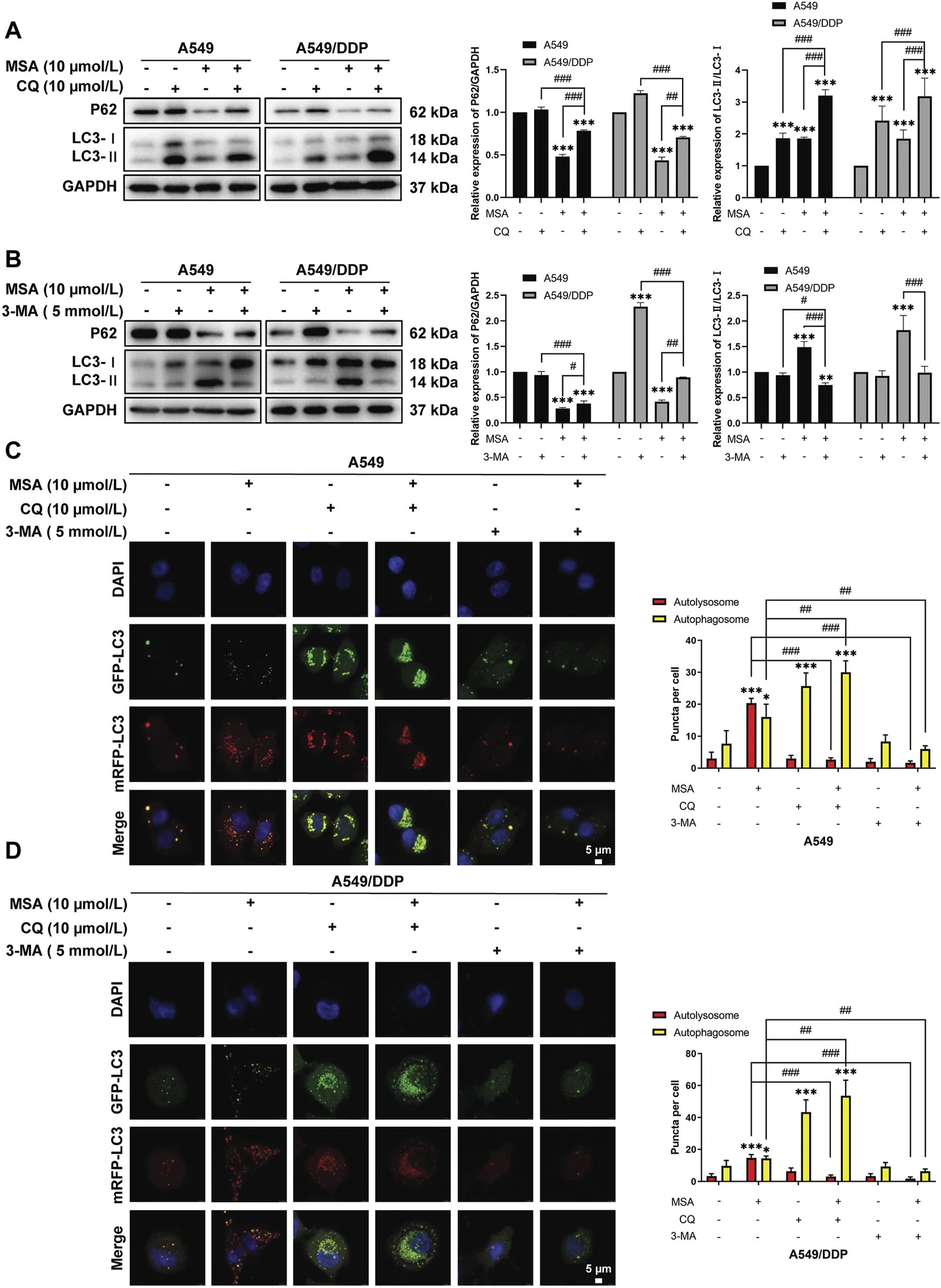
The effect of MSA-induced autophagic flux was blocked by autophagy inhibitors. A549 and A549/DDP cells were treated with MSA (0, 10 μmol/L) in the presence or absence of CQ (10 μmol/L) or 3-MA (5 mmol/L) for 24 h **(A,B)** The protein expression levels of P62, LC3 were detected by western blotting **(C,D)** Observed autophagosomes (yellow puncta) and autophagosomes (red puncta) by CLSM and analyzed LC3 puncta per cell. One representative image is shown. Scale bar: 5 μm *p < 0.05, **p < 0.01, ***p < 0.001 indicated that drug treatment versus control group. #*p* < 0.05, ##*p* < 0.01, ###*p* < 0.001 indicated that CQ or 3-MA combined with MSA treatment versus MSA, CQ or 3-MA single treatment group (*n* = 3).

To further investigate the effect of autophagy on the anti-tumor activity of MSA in cisplatin-resistant LUAD cells, we first tested the inhibition of MSA-induced autophagy by autophagy inhibitors (CQ, 3-MA). Compared with MSA alone, the combined treatment of CQ and MSA increased the expression levels of P62 and LC3-II/I. Conversely, the combination of 3-MA and MSA increased the expression of P62 and decreased the ratio of LC3-II/I **(** Figures 5A,B). These indicate that CQ or 3-MA combined with MSA can inhibit autophagy. In addition, we tested the effect of MSA on the autophagy flux of LUAD cells after the combined use of CQ or 3-MA by CLSM. Due to background autophagy, only a few fluorescent clusters appeared in the control cells. After MSA treatment, the red and yellow fluorescence accumulation spots increased. Since CQ inhibited the combination of lysosomes and autophagosomes, there was no acidic environment, mRFP and EGFP had co-localized, and Merge formed a large number of yellow fluorescent aggregation spots. Compared with MSA alone, the combined treatment of CQ and MSA significantly increased the number of autophagosomes (yellow). It decreased the number of autophagolysosomes (red), similar to the phenomenon observed in the CQ alone treatment group. Compared with MSA alone, the combined treatment of CQ and MSA significantly increased the number of autophagosomes (yellow). It decreased the number of autophagolysosomes (red), similar to the phenomenon observed in the CQ treatment group. The combined treatment of 3-MA and MSA significantly reduced the number of autophagosomes (yellow) and autophagolysosomes (red) **(** Figures 5C,D **)**, similar to the 3-MA treatment group. These proved that CQ or 3-MA combined with MSA could inhibit MSA-induced autophagic flux.

### Inhibition of Autophagy Enhanced the Anti-tumor Activity of Methylseleninic Acid in Cisplatin-Resistant Lung Adenocarcinoma Cells

**FIGURE 6:**
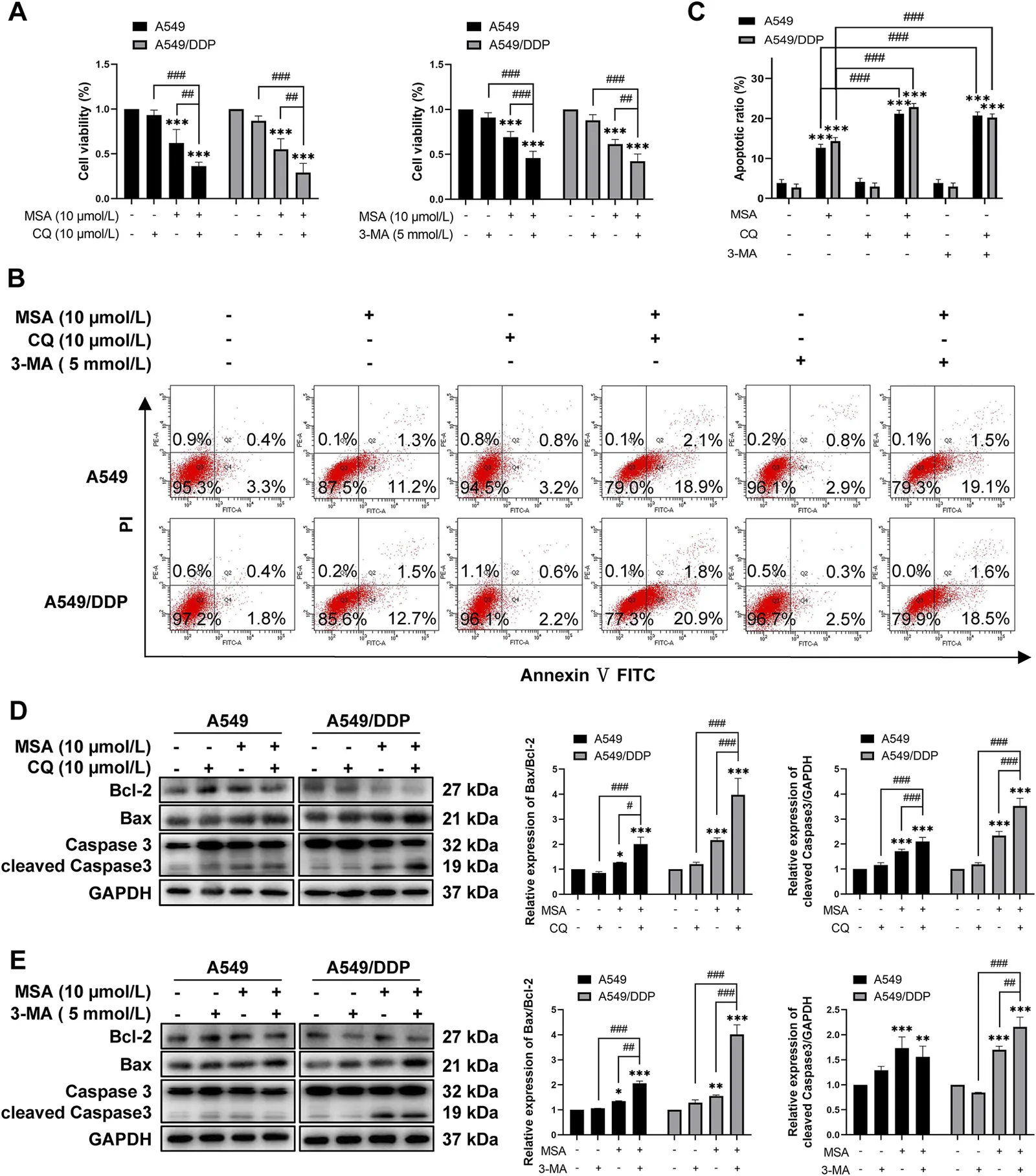
Inhibition of autophagy enhanced the anti-tumor activity of MSA in cisplatin-resistant LUAD cells. A549 and A549/DDP cells were treated with MSA (0.10 μmol/L) in the presence or absence of CQ (10 μmol/L) or 3-MA (5 mmol/L) for 24 h **(A)** The cell viability of MSA was determined by the CCK8 assay **(B)** The rate of apoptosis in the cells was analyzed by flow cytometry **(C)** The apoptotic ratio in **(B)** was quantitated from three independent experiments **(D,E)** The protein expression levels of Bcl-2, Bax, Caspase-3, and cleaved Caspase-3 were measured by western blotting. The data were normalized and used for quantitative analysis and presented as bar graphs. *p < 0.05, **p < 0.01, ***p < 0.001 indicated that drug treatment versus control group and #*p* < 0.05, ##*p* < 0.01, ###*p* < 0.001 indicated that CQ or 3-MA combined with MSA treatment versus MSA, CQ or 3-MA single treatment group (*n* = 3).

After confirming that CQ or 3-MA combined with MSA treatment can inhibit MSA-induced autophagic flux. The anti-proliferative effect of MSA on LUAD cells after autophagy inhibition was examined. Compared with the control group and the single drug treatment group, CQ or 3-MA combined with MSA treatment decreased the viability of A549 and A549/DDP cells **(** Figure 6A). These indicated that the inhibition of autophagy could enhance the anti-proliferative effect of MSA. We also tested the effect of apoptosis by MSA on LUAD cells after autophagy inhibition by flow cytometry. Compared with control and the single drug treatment group, CQ or 3-MA combined with MSA treatment increased the apoptotic ratio of A549 and A549/DDP cells **(** Figures 6B,C), which proved that inhibited autophagy could enhance the apoptosis-inducing effect of MSA. Subsequently, we examined the impact of MSA on the expression of apoptosis-related proteins Bcl-2, Bax, Caspase-3, and cleaved Caspase-3 at the molecular level after autophagy inhibition. The results showed that compared with control and the single drug treatment group, the level of Bax/Bcl-2 and cleaved Caspase-3 were significantly increased after CQ or 3-MA combined with MSA treatment in LUAD cells, except for the changes in the level of cleaved Caspase-3 on A549 cells **(** Figures 6D,E). It has been proved that inhibition of autophagy can enhance the anti-tumor activity of MSA in cisplatin-resistant LUAD cells.

## Discussion

**FIGURE 7:**
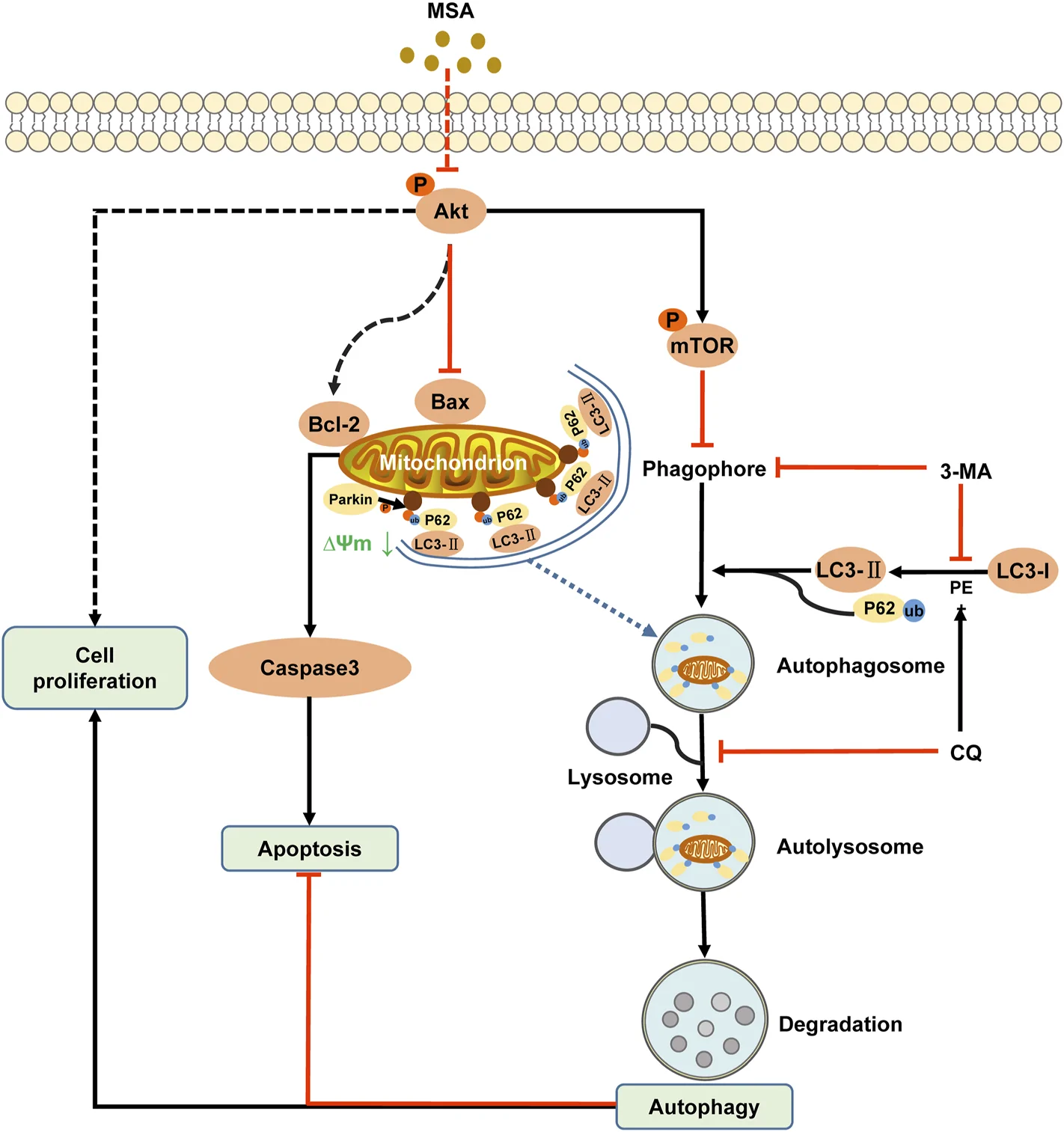
Schematic showing the anti-tumor mechanism of MSA and its effect on autophagy in cisplatin-resistant LUAD cells. MSA can inhibit cell proliferation by inhibiting the phosphorylation of Akt, induce mitochondrial-mediated apoptosis, and promote the death of cisplatin-resistant LUAD cells. At the same time, MSA inhibits the mTOR pathway to induce autophagy against the anti-tumor effect of MSA. Combined with autophagy inhibitors can reduce the resistance caused by protective autophagy.

In the present study, we found that MSA not only has therapeutic potential for cisplatin-resistant LUAD cells, but also induces its autophagy. We speculate that MSA exerted the anti-tumor effect by induced mitochondrial-mediated apoptosis in cisplatin-resistant LUAD cells. Conversely, damaged mitochondria were degraded by inducing selective autophagy in LUAD cell. The combination with autophagy inhibitors reduced the impact of this selective autophagy-induced resistance (Figure 7 **)**.

Currently, platinum-based combination chemotherapy remains the first-line treatment for patients with advanced non-squamous NSCLC (Rizvi et al., 2016; Novello et al., 2017). Unfortunately, its therapeutic efficacy is limited, with an average survival time of only 10 months. The rapid progress of chemotherapy resistance and postoperative metastasis led to treatment failures in most patients. In addition, if the tumor develops cisplatin resistance, it is prone to cross-resistance with other anticancer drugs (Fang et al., 2018). Multidrug resistance protein 1 (MDR1) is a transmembrane transporter responsible for the efflux of anticancer drugs through the cell membrane *via* ATP hydrolysis and its overexpression is crucial in tumor multidrug resistance (Waghray and Zhang, 2018; Kong et al., 2020). Furthermore, cisplatin resistance is associated with cancer stem cells (CSCs), in which ALDH1A1, as a novel CSCs marker, Its enhanced activity has been found identified across a number of cisplatin-resistant NSCLC cell lines and tumor samples (MacDonagh et al., 2018). Its high expression may be related to the expansion of CSCs subsets and the resistance of tumors to broad-spectrum chemotherapy drugs. Therefore, there is an urgent need to find new drugs to improve the treatment of drug-resistant LUAD. In this study, we demonstrated that MDR1 and ALDH1A1 was overexpressed in A549/DDP cells, and drug resistance was shown after treatment with cisplatin in A549/DDP cells. However, MSA treatment of A549 and A549/DDP cells resulted in proliferation inhibition, and increased sensitivity was observed in cisplatin-resistant A549/DDP cells. This suggests that cisplatin-resistant A549/DDP cells overexpressing MDR1 and ALDH1A1 are not resistant to MSA.

The apoptosis of tumor cells is a key indicator of the efficacy of chemotherapy (Friedman et al., 2019). Most conventional chemotherapy and radiotherapy rely on activating the apoptotic pathway to trigger the apoptosis of cancer cells (Yuan et al., 2019). One of the hallmarks of chemoresistance is evading apoptosis (Wendel et al., 2004). The results of flow cytometry analysis showed that MSA could induce apoptosis of A549 and A549/DDP cells in a dose-dependent manner, and MSA treatment at the same concentration can induce more apoptosis of cisplatin-resistant A549/DDP cells. This finding is consistent with the analysis of the TUNEL experiment. It shows that MSA is more sensitive to cisplatin-resistant A549/DDP cells. Apoptosis can be induced by accumulating various pro-apoptotic stimuli on the mitochondria, leading to mitochondrial dysfunction, caspase activation, and ultimately apoptosis (Nagata, 2018). Decrease in MMP is a hallmark event of early cell apoptosis (Jin et al., 2019). The results of JC-1 staining showed that the MMP decreased after MSA treatment, which prevented JC-1 from accumulating in the mitochondrial matrix of A549 and A549/DDP cells. It indicates that MSA may cause apoptosis by affecting the function of mitochondria. In particular, it has been determined that many chemotherapeutics induce tumor cell apoptosis through a mitochondrial-dependent pathway, which is highly regulated by members of the Bcl-2 family (Czabotar et al., 2014). The Bcl-2 protein family is considered to be a critical factor in the regulation of mitochondrial membrane permeability. Its family includes both pro- and anti-apoptotic members, such as Bcl-2 and Bax (Moldoveanu and Czabotar, 2020). The following results showed that after MSA treatment, the overexpression of Bax was accompanied by the down-regulation of Bcl-2, which in turn activated the effector protein Caspase3 that performs the death function, leading to apoptosis in A549 and A549/DDP cells. This apoptosis-inducing effect is more robust on cisplatin-resistant A549/DDP cells. These results well prove that MSA initiates the apoptotic cascade of A549 and A549/DDP cells through the mitochondrial pathway, and the effect of inducing apoptosis is more obvious on cisplatin-resistant A549/DDP cells.

Next, we evaluated the possibility of MSA inducing autophagy. Autophagy participates in the isolation of various cytoplasmic structures and lysosomal degradation, including invading microorganisms and damaged organelles (Shan et al., 2016). Mitochondrial damage leads to a decrease in MMP and at the same time recruits and activates Parkin’s E3 ubiquitin ligase. This causes multiple downstream signaling events to be triggered, ultimately leading to the degradation of damaged mitochondria by autophagosomal phagocytosis (Nguyen et al., 2016). In addition, the recruitment of p62 to polyubiquitin-positive mitochondrial clusters depends on functional Parkin (Geisler et al., 2010). Generally, the expression of p62 is negatively correlated with autophagy activity, and p62 can directly bind to LC3 to promote the degradation of p62 ubiquitinated protein aggregates through autophagy (Pankiv et al., 2007). The results showed that the expression of Parkin increased with the increase of MSA concentration, but it decreased slightly when treated with MSA (20 μmol/L) in A549/DDP cells. This may be due to the increased apoptosis at this concentration, which caused the relative decrease in Parkin protein collection. MSA induced the conversion of LC3-I to LC3-II to be detected, which enhanced autophagy and caused more degradation of p62. It shows that MSA induced autophagy in LUAD cells and may degrade damaged mitochondria through selective forms of autophagy. At the same time, the observed autophagosomes enclosing damaged mitochondria also confirmed this conjecture under the transmission electron microscope (Figure 3C, black arrow). In addition, when treated with MSA, an increase in acidic autophagic vesicles was observed by MDC staining, and an increase in autophagic flux was detected by the localization pattern of mRFP-GFP-LC3. Furthermore, MSA induced less autophagosomes in cisplatin-resistant A549/DDP cells, which may be related to the fact that MSA can induce more apoptosis in A549/DDP cells, but further studies are needed. These results indicate that MSA is indeed induced autophagy in LUAD cells.

Protein kinase B (Akt) is a well-characterized effector of phosphoinositide 3-kinase. Its dysregulation plays a vital role in the proliferation, differentiation, apoptosis, autophagy, and survival of human LUAD. The phosphorylation of Akt at ser473 and thr308 is closely related to the mTOR kinase and its related protein rictor (Atwood et al., 2020; Liu et al., 2021). Autophagy is inhibited by mTOR, which is a core a central cell growth regulator that provides nutritional signals and integrates growth factors (Murugan, 2019). Under diverse cellular stresses, mTOR activity is inhibited from inducing autophagy (Al-bari and Xu, 2020). Our current data showed the expression of p-Akt and p-mTOR in A549 and A549/DDP cells was inhibited by MSA and showed a dose-dependent manner. Furthermore, when the AKT pathway was specifically activated using SC79, the phosphorylation level of mTOR was increased, whereas MSA-induced autophagy was abolished. The same results were obtained when the mTOR pathway was activated using MHY1485. Thus, the results confirmed that MSA induced autophagy *via* the AKT/mTOR signaling pathway, and this pathway may be closely associate with MSA induced apoptosis and inhibited of cell proliferation.

The role of autophagy in malignant tumors is undoubtedly complicated, as it can both inhibit tumor cell growth or promote tumor survival during tumor development. This effect is highly dependent on cell characteristics and survival conditions (Xu et al., 2017). There is still debate whether stress or drug-induced autophagy is protective or causes autophagic death (Zein et al., 2021). Autophagy has been reported to regulate the degradation of LUAD cells, recycle damaged cytoplasmic contents and combat cisplatin-induced DNA damage. It has a facilitative role in protecting LUAD cells from cisplatin mediated anti-tumor effects and inducing cisplatin resistance (Wang et al., 2020). Although MSA has not been proven to be associated with autophagy in lung cancer cells, MSA-induced autophagy in pancreatic adenocarcinoma cell lines appears to be a survival mechanism to counteract the effects of apoptosis in response to MSA exposure (Wang et al., 2014). To verify whether the MSA-induced autophagy in this study is the self-protection mechanism of cisplatin-resistant LUAD cells, we used different stages of autophagy inhibitors in the combined drug intervention. CQ blocks the later stage of autophagy by inhibiting the fusion of autophagosomes and lysosomes, thereby blocking the autophagic flux and inhibiting the degradation of LC3-II. The combination of the two drugs proved that the increase in LC3-II signal of MSA-treated LUAD cells were caused by the formation of autophagosomes, rather than the blocked of autophagy degradation. 3-MA is a PI3K inhibitor that can block the class III PI3Ks to inhibit the early stages of autophagy induced by MSA. This resulted in blocking the autophagic flux from the initial stage and inhibited the conversion of LC3-I to LC3-II. The fluorescence results of mRFP-GFP-LC3 also supported this. After determining that autophagy inhibitors could block MSA-induced autophagic flux, we observed that autophagy inhibition enhanced the anti-proliferative effects of MSA, as well as the promotion of apoptosis. This suggests that autophagy is protective in MSA-treated LUAD cells. These findings indicate that inhibition of autophagy may enhance the anti-tumor effects of MSA on LUAD cells, especially cisplatin-resistant LUAD cells.

Although significant results have been achieved in the present research, there are still several limitations to be considered. First, Few cisplatin-resistant LUAD cell lines were used in this study. More cisplatin-resistant LUAD cell lines should be used to validate the claims of this paper to overcome these differences that are not the result of cloning artefacts. Second, studies demonstrating the potential value of MSA/autophagy inhibitor combination are insufficient. It will be useful to investigate the combination index of MSA/autophagy inhibitors and their effects *in vivo*. Although it is worth mentioning that the lack of such experiments is a limitation of the study, we consider this finding interesting. We plan to continue to construct more cisplatin-resistant LUAD cell lines in the future, as well as to conduct more in-depth studies on the effects of the MSA/autophagy inhibitors combination using animals.

In conclusion, we confirmed that MSA inhibits LUAD cell proliferation and triggers mitochondrial pathway-mediated apoptosis by attenuating the phosphorylation level of Akt, which is more evident especially in acquired cisplatin-resistant LUAD cells. This indicates that MSA has more significant therapeutic potential in cisplatin-resistant LUAD, and it is potentially more valuable when used in combination with autophagy inhibitors.

## Data Availability

The raw data supporting the conclusions of this article will be made available by the authors, without undue reservation.
