# Correction: Autophagy inhibition enhances the anti-tumor activity of methylseleninic acid in cisplatin-resistance human lung adenocarcinoma cells

**DOI:** 10.3389/fphar.2026.1947517

**Published:** 2026-09-23

**Authors:** Ming Xin, Qi Gao, Xindong Xiang, Juanjuan Xu, Yuhan Jiao, Xuan Li, Xianzhen Zhang, Xiuqin Jia

**Affiliations:** 1 The Key Laboratory of Molecular Pharmacology, Liaocheng People’s Hospital, Liaocheng, China; 2 College of Pharmacy, Shandong First Medical University, Taian, China; 3 Department of Oncology, Liaocheng People’s Hospital, Liaocheng, China

**Keywords:** lung adenocarcinoma, cisplatin-resistance, MSA, Akt/mTOR, autophagy, apoptosis

There was a mistake in Figure 2A as published. In the original flow cytometry analysis, the apoptotic ratio was incorrectly calculated as the sum of early apoptotic (Q4, Annexin V^+^/PI^−^) and late apoptotic (Q2, Annexin V^+^/PI^+^) cells. According to standard guidelines for flow cytometric apoptosis measurement (Bio-Rad, ThermoFisher), only Annexin V^+^/PI^−^ (Q4) cells should be counted as true apoptotic cells, as Q2 cells (Annexin V^+^/PI^+^) cannot be reliably distinguished from necrotic cells in this assay. The authors confirm that these corrections do not affect the main conclusions of the study. The corrected Figure 2 appears below.

**FIGURE 2:**
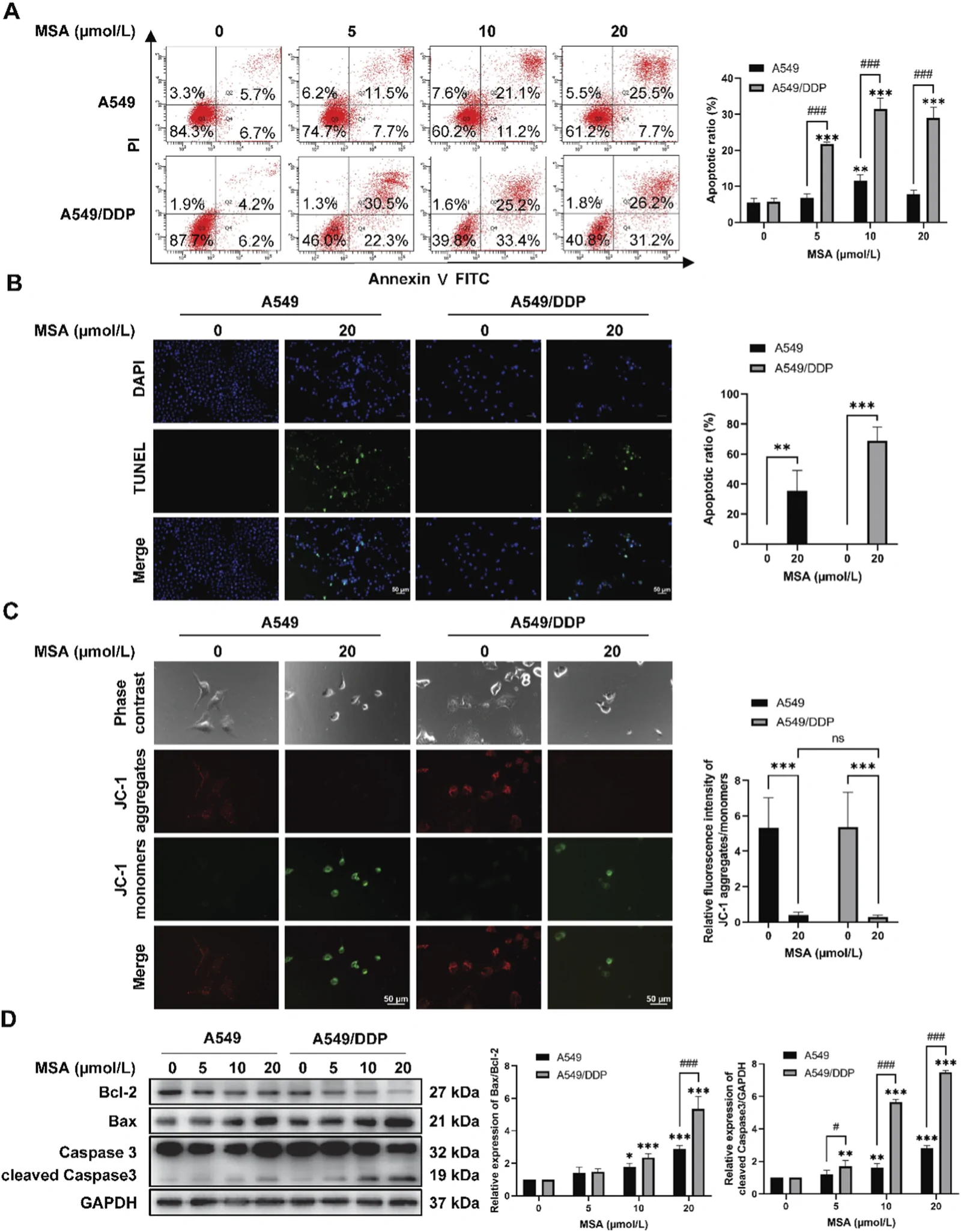
MSA induced mitochondrial-mediated apoptosis in cisplatin-resistant LUAD cells. **(A)** Flow cytometry analysis was used to examine the early apoptotic ratio (Annexin V^+^/PI^−^ cells, Q4) in A549 and A549/DDP cells after MSA treatment. **(B)** Apoptosis was determined by observing and counting TUNEL and DAPI stained cells under fluorescence microscopy. Scale bars: 50 μm. **(C)** Changes in MMP were observed by JC-1 staining under fluorescence microscopy. Scale bars: 50 μm. **(D)** The protein expression levels of Bcl-2, Bax, Caspase-3, and cleaved Caspase-3 were detected by Western blotting, and the relative protein expressions were shown as bar graphs after normalized analysis. *p < 0.05, **p < 0.01, ***p < 0.001 vs. control group (untreated cells), #p < 0.05, ###p < 0.001 indicated that A549/DDP group compared to A549 group under the same concentration of drug treatment (n = 3).

There was a mistake in the caption of Figure 2A as published. The original caption did not specify that the apoptotic ratio was based solely on early apoptotic cells. The corrected caption of Figure 2 appears below.

In the original text, the apoptotic calculation was incorrectly defined as the sum of early apoptotic (Q4) and late apoptotic (Q2) cells.

A correction has been made to the section Materials and Methods, subsection Flow Cytometry Analysis, Paragraph 1:

Original text:

“Apoptosis was calculated as the ratio of early (Q4) and late (Q2) apoptotic cells.”

Corrected text:

“Apoptosis was calculated as the ratio of early (Q4, Annexin V^+^/PI^−^) apoptotic cells. It is noted that late apoptotic cells (Q2) were excluded from the quantification, as they cannot be reliably distinguished from necrotic cells by this assay.”

The original version of this article has been updated.

